# Paraquat and Diquat: Recent Updates on Their Pretreatment and Analysis Methods since 2010 in Biological Samples

**DOI:** 10.3390/molecules28020684

**Published:** 2023-01-10

**Authors:** Honghui Guo, Ling Li, Lina Gao

**Affiliations:** 1 Department of Forensic Analytical Toxicology, China Medical University, Shenyang 110122, China; 2 Department of Forensic Pathology, China Medical University, Shenyang 110122, China; 3 Department of Forensic Genetics and Biology, China Medical University, Shenyang 110122, China; 4 Lianyungang Institute for Food and Drug Control, Lianyungang 222069, China

**Keywords:** paraquat, diquat, sample preparation, analytical methods, review, biological samples

## Abstract

Paraquat (PQ) and diquat (DQ) are quaternary ammonium herbicides which have been used worldwide for controlling the growth of weeds on land and in water. However, PQ and DQ are well known to be toxic. PQ is especially toxic to humans. Moreover, there is no specific antidote for PQ poisoning. The main treatment for PQ poisoning is hemoperfusion to reduce the PQ concentration in blood. Therefore, it is essential to be able to detect PQ and DQ concentrations in biological samples. This critical review summarizes the articles published from 2010 to 2022 and can help researchers to understand the development of the sample treatment and analytical methods for the determination of PQ and DQ in various types of biological samples. The sample preparation includes liquid–liquid extraction, solid-phase extraction based on different novel materials, microextration methods, and other methods. Analytical methods for quantifying PQ and DQ, such as different chromatography and spectroscopy methods, electrochemical methods, and immunological methods, are illustrated and compared. We focus on the latest advances in PQ and DQ treatment and the application of new technologies for these analyses. In our opinion, tandem mass spectrometry is a good choice for the determination of PQ and DQ, due to its high sensitivity, high selectivity, and high accuracy. As far as we are concerned, the best LOD of 4 pg/mL for PQ in serum can be obtained.

## 1. Introduction

**Figure 1:**
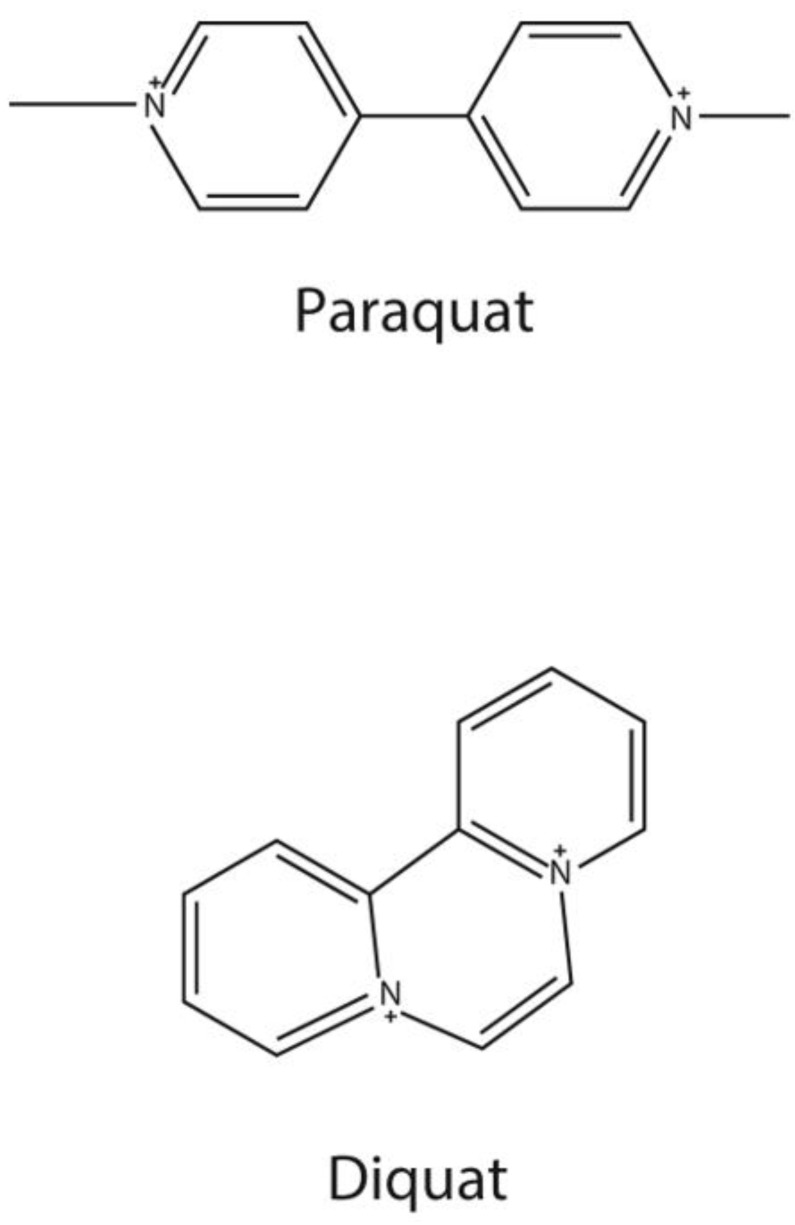
Chemical structure of paraquat and diquat.

Paraquat (PQ) and diquat (DQ) (Figure 1) are bipyridylium herbicides. They are non-selective contact herbicides [1] and are used worldwide. However, PQ is highly toxic to humans, and it is a common poison in suicide and accidental poisoning [2]. In the early stage, PQ poisoning can cause acute lung injury or acute respiratory distress syndrome, and most patients die of multi-organ dysfunction syndrome (MODS) or respiratory failure, whose pathogenesis is still unclear [3,4]. At present, there is no specific detoxification drug for paraquat poisoning, and the clinical treatment is still under exploration. At present, removing paraquat from the body is the main way to treat patients with acute paraquat poisoning [5]. Moreover, serum/plasma PQ concentration was used to assess the prognosis of PQ poisoning [2,5,6,7]. In general, the serum concentrations of patients do not exceed 2.0, 0.6, 0.3, 0.16, and 0.1 μg/mL at 4, 6, 10, 16, and 24 h after ingestion, respectively, and they are likely to survive [8,9].

In recent years, as PQ has been banned in China, DQ has rapidly taken over the market, but at the same time, the number of poisonings due to DQ herbicide has also increased dramatically. Moreover, the vast majority of the products sold under the name “Diaquat” are still “paraquat” [10].

In summary, the ability to conduct qualitative and quantitative analyses of DQ and PQ quickly and accurately is important for clinical treatment. In 2023, Rajaram et al. [11] summarized the various analytical techniques for PQ detection in food samples and environmental samples. However, as there are more interferences in the biological samples than there are in the non-biological samples, the developed methods for non-biological samples are not suitable for biological samples detection. As PQ and DQ are both highly soluble in water and have an extremely high polarity, it is often problematic to conduct the separation and preconcentration before the detection of PQ or DQ. However, there are no comprehensive reviews about sample preparation and determination methods in biological samples, even though the methods for the determination of PQ and DQ have been well developed. In this paper, we provide a summary of the purification and determination methods for PQ and DQ in different biological samples reported from 2010 to 2022. Moreover, this review lists the advantages and drawbacks of different sample preparation and analytical methods and presents the development trends.

## 2. Sample Treatment Methods

Owing to the low concentration (µg/mL) of PQ and DQ in complex biological samples, a sample preparation method is necessary for the effective extraction of analytes and the removal of interference [12]. The analysis efficiency and sensitivity can be improved by using an appropriate sample treatment method before instrument testing. In this review, recent advances in sample preparation methods for PQ and DQ analysis are discussed. The sample treatment methods for PQ and DQ (Table 1) include protein precipitation [12,13,14,15,16,17,18,19], liquid–liquid extraction (LLE) [20,21], solid-phase extraction (SPE) [22,23,24,25,26,27], solid-phase microextraction (SPME) [28,29], liquid-phase microextraction (LPME) [10,30,31], and magnetic dispersed solid phase extraction (MDSPE) [32,33,34,35,36].

### 2.1. Protein Precipitation

Trichloroacetic acid can be used as a protein precipitant for the preparation of paraquat-containing biological samples [17], but it was abandoned because of its low pH value, which would damage the column and reduce the column efficiency. In recent years, acetonitrile has been used more as a paraquat and diquat protein precipitant, and a good extraction efficiency has been achieved. Wunnapuk et al. [20] used one-step protein precipitation with cold acetonitrile to prepare paraquat samples from plasma and urine. Other experiments also used an acetonitrile protein precipitation method in the preparation of biological samples of paraquat and diquat [21]. Lu et al. [13] found that, compared with double acetonitrile precipitation, triple acetonitrile precipitation achieved better extraction and purification, and the extraction recovery rate of paraquat in plasma and urine was 80% and 90%, respectively. The advantages of one-step acetonitrile protein precipitation are its simplicity, short preparation time, and lack of enrichment steps. However, this sample treatment does not take into account the interaction between the sediment and the paraquat. Because the sediment is disposed, the concentration of analytes may be underestimated.

### 2.2. Liquid–Liquid Extraction (LLE)

LLE is a sample treatment technique that was used more in the past to isolate and extract analytes. As LLE is tedious and not environmentally friendly, it has been gradually replaced by solid phase extraction. In the past ten years, only a few articles report using LLE to enrich paraquat and diquat in biological samples [21]. However, Baeck et al. [37] compared SPE and LLE procedures in PQ analysis in post-mortem human blood and found that LLE can obtain satisfactory recovery. In summary, this treatment method is complicated and time-consuming, and it is not optimal for the rapid analysis of paraquat in clinical diagnosis.

### 2.3. Solid-Phase Extraction (SPE)

SPE, developed in the 1970s, has replaced traditional liquid–liquid extraction and has become an effective means of sample treatment in many fields because of its high efficiency, reliability, lower reagent consumption, and other advantages. However, ordinary SPE reagents such as C18 cartridges and HLB are not suitable for the extraction of PQ and DQ, which is directly due to their solubility and strong polarity. The Oasis WCX solid-extraction column was suitable for capturing paraquat and diquat from plasma samples, and the extraction recovery was more than 90% [22,24,25]. Compared with C18 cartridges, this kind of cation exchange column was more suitable for the treatment of high-polarity, ionizable samples, and it had advantages including a small sample size, high extraction recovery, and good reproducibility.

In recent years, modified solid-phase extraction methods have been developed, such as monolithic spin column extraction and MDSPE. Saito et al. [26] developed a simple, sensitive, and specific method for the detection of PQ and DQ in human serum and urine. First, PQ and DQ derivatize with sodium borohydride; then, they extract the derivatization by using a monolithic silica spin column. All the steps, including loading, washing, and elution, are completed by centrifugation alone. After optimization, the recoveries were in the range of 67–94% and 72–97% for PQ and DQ, respectively.

MDSPE has been a revolutionary technology in the field of separation and enrichment in the 21st century. MDSPE is a dispersible solid-phase extraction technology using magnetic or magnetizable materials as adsorbent substrates. Compared with conventional SPE, MDSPE has a very high extraction capacity and extraction efficiency and has been increasingly used in the separation and preconcentration of PQ and DQ [32,33,34,35,36]. In contrast to traditional SPE, MDSPE requires the preparation of a magnetic sorbent for paraquat extraction from biological samples, and filtration or centrifugation is replaced by magnetic separation. Different sorbent materials were developed, including an Fe3O4@SiO2 adsorbent [36], CoFe2O4@SiO2 magnetic nanoparticles [35], benzenesulfonic acid group-modified magnetic microspheres [33], magnetic single-walled carbon nanotubes (MSWCNTs) [34], and amphiphilic carboxyl-functionalized magnetic polymer microspheres [32]. Sha et al. [35] developed an efficient extraction analysis from human plasma and urine samples using CoFe2O4@SiO2 NPs solid phase extraction. The main parameters affecting the extraction efficiency included the amount of extractant, the extraction time, the sample volume, the sample solution pH, and the elution volume. After optimization, the recoveries were in the range of 88–99%. Sha et al. [36] developed Fe3O4@SiO2 NPs solid-phase extraction combined with UV-Vis, and high recoveries (ranging from 93% to 105%) have been obtained.

### 2.4. Microextraction Methods

Based on the need to develop environmentally friendly extraction methods, liquid-phase microextraction (LPME) and solid-phase microextraction (SPME) were developed.

Liquid-phase microextraction (LPME) was first proposed by Jeannot and Cantwell in 1996 as a new micro-sample treatment technology [10] which uses different distribution ratios of substances in two immiscible phases to achieve separation. LPME includes single-drop microextraction (SDME) [31], hollow-fiber-protected liquid-phase microextraction (HF-LPME) [38], and dispersive liquid–liquid microextraction (DLLME) [39]. In recent years, an ion pair switchable hydrophilic solvent-based homogeneous liquid–liquid microextraction (SHS-LLME) method has been applied to preconcentrate paraquat in environmental and biological samples [12,30,40,41]. Compared with traditional extraction technology (such as LLE and SPE), LPME has the advantages of a simple operation, high enrichment times, a strong selectivity, and environmental friendliness, and it can be used in conjunction with high-performance liquid chromatography (HPLC), gas chromatography (GC), mass spectrometry (MS), and other analytical instruments. Kumari et al. [31] applied a whirling agitated SDME for the determination of PQ in tissue samples, and the limit of detection of PQ was 4.81 ng/g. The mean recoveries and enrichment factors obtained were >91% and up to 114, which demonstrated the effectiveness of this method. Kakavandi et al. [30] developed an ultrasound-assisted SHS-HLLME combined with GC–MS for the detection of PQ in biological and environmental samples. With this method, there were 230 enrichment factors for water and apple juice samples and 150 for biological samples.

SPME integrates extraction, preconcentration, and sampling into one, which is simple, rapid, economical, safe, solvent-free, selective and sensitive. Headspace solid-phase microextraction (HS-SPME) is suitable for the detection of volatile or semi-volatile components in a gaseous, liquid, or solid sample. Gao et al. [29] used HS-SPME as a sample treatment method after the derivatization of PQ, and a 100 μm polydimethylsiloxane coating was selected to absorb the analyte. After optimization, the recoveries in plasma and urine samples were 94–100% and 95–100%, respectively.

### 2.5. Other Treatment Methods

Di Wen et al. [42] developed a dried blood spot (DBS) method for extracting paraquat from human blood. Several droplets of the whole blood sample were deposited on the Whatman^®^ FTA classic card and irradiated by a commercial microwave for 5 min until completely dry. When analysis was needed, the sample area of the DBS was cut and placed in a tube with 190 μL of alternative extraction solvent for 10 min by ultrasound. The main advantage of the DBS method is that it allows for the very simple treatment of the sample and the transportation of the sample.

### 2.6. Summary

Due to the characteristics of PQ and DQ, such as their solubility in water and strong polarity, there are many problems in their treatment and analysis. The complexity, time consumption, and equipment requirements of most methods make them impractical, especially in biological samples. Improved sample preparation methods for extracting PQ or DQ from plasma and urine samples, such as SPE and LLE, have been developed. However, the complexity of the process, including the acetonitrile precipitation of proteins or the removal of proteins using ultrafiltration membranes, on the one hand reduces the sensitivity of the assay and on the other hand increases the analysis time [43]. The improved solid-phase extraction has a high potential utility. MDSPE is a particularly promising technique for sample separation and treatment because it significantly reduces the sample preparation time and avoids the loss of trace analytes as the matrix proteins precipitate.

## 3. Analytical Methods

**Figure 2:**
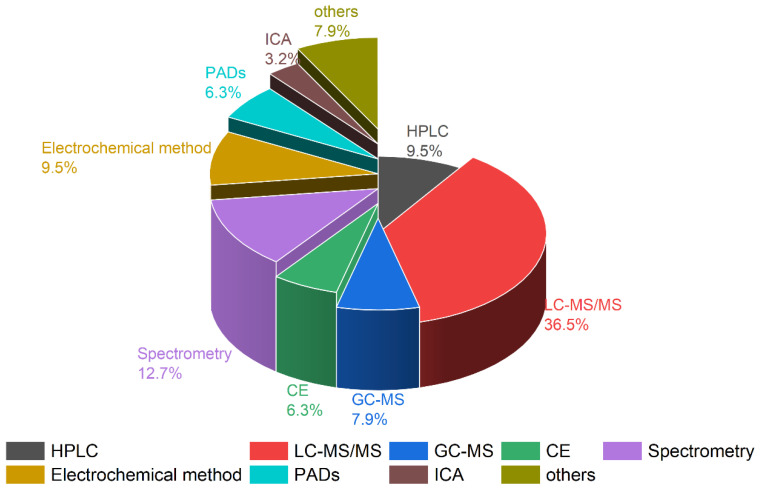
Comparison of the different analytical methods for paraquat and diquat in biological samples.

For the accurate qualitative and quantitative concentration determination of paraquat and diquat, various analytical methods have been developed, including high-performance liquid chromatography–ultraviolet detection (HPLC–UV) [15,18,21,30,35,44,45,46,47], gas chromatography–mass spectrometry (GC–MS) [26,28,29,48], liquid chromatography–mass spectrometry (LC–MS/MS and LC–HRMS) [13,20,25,27,32,34,49,50,51,52,53,54,55], capillary electrophoresis (CE) [56,57,58], surface-enhanced Raman spectroscopy (SERES) [59,60,61], and electrochemical methods [62,63,64,65], which we summarize in Figure 2.

### 3.1. Liquid Chromatography

PQ and DQ are typically separated using LC because they are both highly soluble in water and have an extremely high polarity. In the LC method, the mobile phases, chromatogaphic columns, additives, and column temperature are among the important variables that should be optimized in order to obtain satisfactory chromatographic separation. According to the literature, reverse-phase columns, especially C18 columns and HILIC columns, are most frequently used for PQ and DQ.

#### 3.1.1. High-Performance Liquid Chromatography

High-performance liquid chromatography (HPLC) is most commonly used for the determination of paraquat and diquat because it is common in the laboratory and inexpensive compared with GC–MS or LC–MS, and it has a higher sensitivity and separation efficiency. Sha et al. [30] proposed a method for the determination of PQ and DQ in biological samples using HPLC-UV. First, MDSPE was performed for paraquat and diquat. After phosphoric acid elution, an HPLC-UV system was used for the separation and detection of paraquat and diquat at wavelengths of 258 nm and 310 nm, respectively. Finally, the detection limits of paraquat and diquat were 4.5 ng/mL and 4.3 ng/mL, respectively. Shindo et al. [66] established an HPLC coupled with a chemiluminescence (CL) detection system, and the limit of detection (S/N = 3) was 40 nM for paraquat and 53 nM for diquat. Merritt et al. [42], using UHPLC with photodiode array detection to detect PQ, obtained a rapid (3 min) assay of an organism sample. Zou et al. [21] developed an HPLC method using an ion pair reagent, which acts as the mobile phase, and the analytes were separated on an Xtimate C18 column. The overall recovery of this method was 97.6–107.3%, and the lower limit of detection was 0.01 μg/mL.

#### 3.1.2. Liquid Chromatography–Mass Spectrometry (LC–MS)

At present, LC–MS is widely used in the field of drug toxicology analysis, and it has become one of the important methods for the separation and identification of compounds. Of course, there has been much literature on using LC–MS to detect paraquat or both paraquat and diquat in biological samples. Heptafluorobutyric acid (HFBA) as an ion pair was beneficial in enhancing the retention of the charged substances paraquat and diquat in the chromatographic column [19]. However, these ion-pair regents can reduce the sensitivity of the mass spectrometry detection and introduce additional impurities to the mass spectrometry system. Because PQ and DQ are highly polar, ionic compounds, conventional reversed-phase liquid chromatography columns are not suitable for retaining and separating these two compounds. Hydrophilic interaction liquid chromatography (HILIC) is another method for separating highly polar compounds, which can not only improve the retention of polar substances but also enhance the signal response of mass spectrometry [20,32,33]. The main factors affecting HILIC retention behavior are the flow rate, the column temperature, the pH value of the buffer salt system in the mobile phase, and the type and concentration of buffer salt. Considering that PQ and DQ are doubly charged cationic substances, some researchers have separated them by cation exchange chromatography [22]. Some examples of liquid chromatography–tandem mass spectrometry for the detection of paraquat and diquat are listed in Table 2.

In MS detectors, the ion source is important and affects the ionization efficiency of analytes [67]. Electrospray ionization (ESI) has been used in LC–MS for the detection of PQ and DQ [13,20,22,25,27,29,32,34,49,50,53,55,68]. Yoshioka et al. [54] utilized atmospheric pressure photoionization (APPI) as an ionization technique to detect PQ and DQ in human serum rapidly and sensitively, combined with the liquid chromatography/time-of-flight mass spectrometry (LC/TOF–MS) method. The LODs of PQ and DQ in human serum were 0.005 and 0.006 μg/mL, and the LOQs were 0.015 and 0.021 μg/mL, respectively.

The mass analyzer is also an important part of the MS detector, which can substantially affect the performance of the instrument, including its resolution, sensitivity, and selectivity. At present, single quadrupole MS is seldom applied in the determination of PQ and DQ due to its low resolution. Compared with single quadrupole MS, triple quadrupole (QqQ) MS has been used more and more because of its higher sensitivity, better selectivity, and unequivocal identification of the analytes [20,22,49,50,55]. Although the triple quadrupole is a good quantitative tool, its resolution needs to be improved. Recently, high-resolution mass spectrometry (HRMS) has attracted increasing attention for its high resolution (>10,000) [13,32,51,54].

Ultra-high-performance liquid chromatography–mass spectrometry (UHPLC–MS) is a relatively new detection and analysis technology developed on the basis of HPLC–MS. It has significant advantages such as a high sensitivity, fast analysis speed, and high-resolution separation. There are a few papers that applied UHPLC–MS for the detection of PQ and DQ in biological samples [13,32,42,69,70]. Lu et al. obtained LODs of 0.1 and 0.3 ng/mL and LOQs of 0.3 and 0.8 ng/mL for urine and plasma by UPLC–ESI–HRMS/MS, respectively [13]. Pan et al. [32] combined a treatment method of MDSPE with UPLC–HRMS to obtain LODs and LOQs in the range of 0.1–1.6 μg/L and 0.3–4.8 μg/L, respectively. Wen et al. [42] developed a novel method for the detection of PQ utilizing dried blood spot (DBS) extraction and UHPLC–HRMS, affording an LOD of 0.5 ng/mL and an LLOQ of 1 ng/mL.

Commonly used HRMS detectors include orbitrap MS, ion trap, time-of-flight mass spectrometry (TOF MS), and FTICR. FTICR/MS has an extremely high resolution, but its high data acquisition speed limits its on-line coupling with UPLC [68,69,70,71]. Time-of-flight mass spectrometry has innate performance advantages over quadrupole mass spectrometers. TOF MS captures instantaneous full mass scan information, greatly improving the speed and sensitivity of the instrument analysis, ensuring that no important information is lost, allowing for backtracking analysis, and making it easier to identify unknown analytes. More importantly, the high-quality resolution and precision of TOF MS are more conducive to the accurate identification of unknown species in complex substrates [51,54].

Fan et al. [53] established a method combined with electrospray quadrupole linear ion trap MS (LIT–MS) for detecting PQ in urine and plasma samples. Ion trap mass spectrometry is a convenient method for multistage mass spectrometry analysis, which is very useful for molecular structure identification. Moreover, the resolution of ion trap with a full mass scan mode is higher than that of TOF and quadrupole mass spectrometry. However, its quantitative ability is not as good as that of quadrupole mass spectrometry.

The orbitrap analyzer has the same advantages as TOF and the ion trap, such as an outstanding resolution and mass analysis speed, and it can used to quantify unknown compounds [13,72,73,74]. Table 3 shows the merit and demerit of the MS detectors. Yang et al. [19] developed a sensitive orbitrap HRMS method for the detection of common herbicides in blood. The LOD and LOQ of this method were 5–10 ng/mL and 10–20 ng/mL, respectively. Pan et al. [33] proposed a sensitive orbitrap HRMS method combined with MDSPE for the detection of PQ and DQ in biological samples. A heated electrospray ionization source (HESI) in the positive mode was utilized in the HRMS system. The instrument was operated in the parallel reaction monitoring (PRM) mode, and the MS2 resolution was set at 17,500 FWHM (full width at half maximum). The LODs of PQ and DQ were 0.12 μg/L and 0.14 μg/L, while the LOQs were 0.36 μg/L and 0.42 μg/L, respectively.

HRMS has the advantages of a high mass spectrum resolution, a fast scanning speed, accurate isotopic abundance information, and a wide *m*/*z* dynamic scanning range that can supply accurate qualitative information. Moreover, HRMS has a unique advantage for screening unknown compounds, and its use in the field of metabolomics has become popular [51,69,70,74].

### 3.2. Gas Chromatography–Mass Spectrometry (GC–MS)

GC–MS is widely used in the analysis of complex components and has the advantages of a high resolution and high sensitivity. Paraquat and diquat have a high polarity and low volatility. PQ, on account of its non-volatile, cationic compound, is not suitable for analysis by GC [12,30]. To detect PQ and DQ in biological samples by GC–MS, it is necessary to convert them into thermally stable and volatile substances prior to sampling [12,29,48]. Gao et al. [29] developed an HS–SPME–GC/MS methodology for quantifying PQ concentrations in plasma and urine samples. Sodium borohydride–nickel chloride (NaBH4–NiCl2) was used to reduce paraquat in biological samples, and HS-SPME was used to extract the reduced paraquat and internal standard EPQ. With this method, the LOD was 10 ng/mL in plasma and urine samples. In general, the operation of GC–MS is relatively complex and time-consuming and often requires derivative processing, so it is seldom used in practical applications. Navid et al. [12] developed UA–SHS–HLLME coupled with GC–MS to detect PQ in various samples including biological and food samples, and the LODs and LOQs obtained were in the range of 0.06–0.13 and 0.20–0.30 ng/mL for GC–MS, respectively. Moreira et al. [48], using GC–ion trap mass spectrometry after SPE, obtained LODs ranging from 0.0076 µg/mL to 0.047 µg/mL for urine and whole blood, respectively.

In recent years, GC–MS has not only been applied to determine the concentration of PQ and DQ in biological samples but has also been coupled with high-resolution mass spectrometry (HRMS) to study the mechanism of paraquat poisoning, including by Ni et al. [75] and Yu et al. [74], using GC/TOF–MS for metabolomics from paraquat intoxicated mouse models.

### 3.3. Capillary Electrophoresis (CE)

Capillary electrophoresis technology is driven by a high-voltage direct-current electric field. The components in the capillary are separated according to their charge, molecular size, isoelectric point, and other characteristics. It has a high separation efficiency and can afford unique advantages in the separation of small molecules [56,57,58,71]. Lanaro et al. [57] developed capillary electrophoresis (CE) combined with diode-array detection (DAD) to monitor PQ concentration in different biological samples including plasma, urine, and oral fluid. CE–DAD analysis was performed in fused-silica capillaries, using a 40 mM phosphate buffer solution at pH 2.50 as the electrolyte. The separation and analysis were performed at a constant voltage of +21 kV and a detection wavelength of 195 nm. The CE–DAD method had a recovery range of 83% to 109%, and the LOD and LOQ of this method were 50 ng/mL and 100 ng/mL, respectively. This method has been successfully applied to the diagnosis of acute paraquat poisoning. Recently, Anh et al. [56] monitored the PQ concentration in plasma samples using CE with capacitively coupled contactless conductivity detection (C4D). After the enrichment of paraquat by SPE, the obtained solution was injected into the separation capillary. Paraquat was separated by electrophoresis at +20 kV. The optimized background electrolyte was composed of 10 mM histidine and adjusted to pH 4 with acetic acid. The CE-C4D system has a high versatility, is easy to build and operate at a low cost, and can reach a detection limit of 0.5 μg/mL.

### 3.4. Elcetrochemical Sensors

Among the various methods for the determination of paraquat and diquat, there are liquid chromatography, liquid chromatography–tandem mass spectrometry, capillary electrophoresis, spectroscopy, SERES, etc., but all of them require time-consuming sample preparation and expensive instruments and cannot achieve the requirements of rapid on-site detection. Electrochemical sensors have attracted much attention due to their high detection speed, low cost, high sensitivity, and simple preparation, which make it possible to be miniaturized and allow for portability [76]. Metal electrodes and carbonaceous electrodes are the main types in the electrochemical detection of PQ and DQ, with the former electrode incorporating gold electrodes [63,77] and the latter electrode including glassy carbon electrodes (GCEs) [65] and carbon paste electrodes [78,79], as shown in Table 4.

Niu et al. [77] modified gold electrodes with unmodified DNA molecules with consecutive adenines (CA DNA) and gold nanoparticles (GNPs). In this work, the detection limit of it is 2.0 × 10^−10^ mol/L for DQ. Sun et al. [80] utilized novel materials—fullerene, ferrocene, and the ionic liquid—to modify a glassy carbon electrode, and an LOD of 9.0 × 10^−12^ mol/L for PQ was obtained. El Mhammedi et al. [78] reported that FAP (fluorapatite) has been used to modify CPE (carbon paste electrode). Usually, for electrochemical sensors, the important procedure is the making of the modified sensor, where the accumulation time, novel materials loading, and solution pH should be investigated. After the optimization for working conditions, there is a good linearity in the range of 5 × 10^−8^ to 7 × 10^−5^ mol/L, with an LOD of 3.5 × 10^−9^ and 7.4 × 10^−9^ mol/L, respectively. Abu Shawish [79] used 2-nitrophenyloctyl ether (2-NPOE) to modify a carbon paste electrode. There is a good linearity in the range of 3.8 × 10^−6^ to 1.0 × 10^−3^ mol/L, with an LOD 9.0 × 10^−7^ mol/L.

### 3.5. Surface-Enhanced Raman Spectroscopy (SERS)

In the past few years, surface-enhanced Raman spectroscopy (SERS) has emerged as a technique for the determination of paraquat in biological samples [59]. Zhu et al. [61] developed a highly sensitive and fast SERS method for the detection of PQ in urine and plasma samples using a portable Raman spectrometer, with an LOD of 1 ng/mL. In this work, the entire analysis was completed within 1 min without treatment. Yao et al. [81] developed a simple SERS sensor modified by Ag nanoparticles (NPs) that can generate many hot spots for SERS enhancement. When the sensor was applied to determine PQ, there was a good linearity between the SERS signal intensity of PQ and its concentration, and the detection range was wide, from 5 ng/mL to 50,000 ng/mL. The limit of detection was 1.2 ng/mL. The method had high reproducibility. Qin et al. [60] reported an improved SERS method using an internal standard to correct the results for the determination of PQ in plasma and lung tissue, which performed one test every 10 s. They also performed protein precipitation, microdialysis, and plasma fractionation experiments to test for the underestimation of free paraquat concentrations during the treatment of common biological samples. With this method, the LOD and LOQ of PQ were 0.5 μg/L and 0.1 μg/g (plasma sample) and 5 μg/L and 1 μg/g (lung sample), respectively. The improved internal standard–SERS (IS–SERS) method effectively solved the problem of uneven SERS hot spots and effectively improved the accuracy and reproducibility of SERS. This method has a high sensitivity and high-throughput feasibility, but SERS is rarely used in remote areas and clinical laboratories.

### 3.6. Immunochromatographic Assay (ICA)

An enzyme-linked immunosorbent assay (ELISA) was used to quantify paraquat in urine samples, but it was prone to false-positives. Zhang et al. [82] performed a time-resolved fluorescence immunochromatographic assay (TRFICA) strip for the detection of paraquat, using nanobody-based time-resolved fluorescent microspheres as the signal probe. The method was highly sensitive for paraquat, with an LOD of 0.0090 ng/mL. In practical applications, the recovery range of this method is 76.7% to 133.3%. The method is convenient and rapid and can complete a detection in 8 min without cross-reaction with paraquat analogs.

In addition, Fu et al. [83] developed a novel naked-eye immunochromatographic strip for PQ to detect PQ rapidly in different samples including water, urine, and plasma samples. The total analytical time was less than 10 min, and the LOD for PQ was 10 ng/mL, which was comparable to that of TOF MS detection. It can be used for trace detection PQ in a mixture of DQ and PQ.

### 3.7. Paper-Based Analytical Devices (PADs)

More recently, paper analytical devices have become a diagnostic tool for the point-of-care testing of paraquat [80]. Paper analysis devices employ a colorimetric assay after sodium dithionite derivatization to determine serum paraquat levels in patients with paraquat poisoning in 10 min [84]. PQ reacts with sodium dithionite to form a blue radical ion. Some researchers have also developed two-in-one PADs that simultaneously determine serum paraquat and creatinine levels to quickly detect paraquat poisoning and assess renal prognosis [85]. With this method, the LOD and LOQ of PQ were 3.01 μg/mL and 10.02 μg/mL, respectively. The PAD was comparable to that of other conventional colorimetric methods, and the urine paraquat concentration detection was non-invasive and had prognostic significance [85].

### 3.8. Other Analytical Methods

Besides the various analytical methods mentioned above, there are also UV-Vis spectrophotometry methods. Sha et al. [31] used Fe3O4@SiO2 nanoparticles (NPs) as the MSPE materials, and the paraquat absorbed on the NPs was eluted using the eluent of NaOH and ascorbic acid. This method can analyze trace levels with an LOD of 12.2 μg/L. Li et al. [86] developed a method using second-derivative spectrophotometry to determine the PQ concentration in a serum sample. In the range of 0.4–8.0 μg/mL, there is a good linearity (r = 0.996), and the lower detection limit is 0.05 μg/mL. de Almeida et al. [87] developed an enzymatic-spectrophotometric method based on the velocity of NADPH consumption for the detection of PQ and obtained an LOD of PQ of 0.05 μg/mL in urine samples. Li et al. [88] developed a resonance light scattering (RLS) quenching technique with a spectrofluorometer to detect PQ in urine sample. The calibration curve was in the range of 0.05–1.0 μg/mL, and the LOD was 0.036 μg/mL for PQ.

Sun et al. [89] developed a fluorescence enhancement method to detect PQ in living cells and live mice. In this work, PQ easily derivatived with cucurbit (CB [8]), yielding PQ@CB. The calibration curve was in the range of 2.4 × 10^−10^ M–2.5 × 10^−4^ M, and the detection limit was 2.4 × 10^−10^ M (0.06 μg/L) for PQ.

Usui et al. [68] used probe electrospray ionization–tandem mass spectrometry to detect PQ in serum; the LOD and LOQ were 0.004 and 0.015 μg/L, respectively, and there was a good linearity in the range of 0.015–4.0 μg/mL for the PQ concentration.

Chen et al. [90] developed MALDI–FTICR–MS to determine the PQ in the whole blood and urine samples. MALDI–FTICR–MS has advantages including an ultra-high resolution, high sensitivity and accuracy, a low sample consumption, a convenient preparation, speed, and an ease of operation [91,92,93], as shown in Table 3. It is suitable for the direct and rapid determination of trace components in complex biological samples. The optimization conditions of MALDI sources are usually the accumulation times, the laser energy, the laser frequency, the number of laser points, and the spot size. Among these, the accumulation times have the greatest influence on the peak intensity of paraquat ions [90]. The limits of detection in whole blood, urine, and water were 3.0, 1.5, and 0.6 μg/L, and the limits of quantification were 10, 5, and 2 μg/L, respectively.

Chen et al. [71] developed a microcapillary sampling (MCS) method coupled with matrix-assisted laser desorption/ionization Fourier transform ion cyclotron resonance mass spectrometry (MALDI–FTICR–MS), which was used to detect the PQ concentrations in living vegetables. After the optimization of the parameters, there was a wide linear range (7.81–500 μg/kg) and a low limit of detection (0.1–0.9 μg/kg) for PQ and DQ.

Tan et al. [94] reported that surface-assisted laser desorption/ionization time-of-flight mass spectrometry (SALDI-TOF MS) was utilized to analyze paraquat (PQ) and diquat (DQ), where sulfonic acid-functionalized hierarchical porous covalent organic frameworks (H-COF-SO3H) were used as the matrix and adsorbent for the detection of PQ and DQ. The LODs for PQ and DQ with H-COF-SO3H enrichment were 0.5 and 0.1 ng/mL. Although MALDI or SALDI are mostly used in the food chemistry and environmental fields, we believe that they will be applied in biological samples in the future for their unique advantages.

### 3.9. Summary

At present, the detection methods for PQ and DQ mainly include GC–MS, HPLC, LC–tandem MS, CE, SERS, and ELISA. Among these, LC is the most commonly used detection method for PQ and DQ due to its simplicity, ease, low cost, and reproducibility. It can be applied to the rapid detection of the blood drug concentration in clinical patients with paraquat poisoning. However, it has disadvantages such as a low sensitivity and poor ability to identify unknown analytes. The most commonly used LC columns are C18 and HILIC. The poor sensitivity of CE limits its application in clinics. The robustness and accuracy of SERES need to be improved. When ELISA was used to quantify paraquat in urine samples, it was prone to false-positives. GC–MS is common and has a high sensitivity, but PQ and DQ require derivatization before analysis. As a widely used technology in recent years, ultra-high-performance liquid chromatography–tandem mass spectrometry has the characteristics of high sensitivity, trace detection, and accurate quantitative analysis, and it has become an important means of detection and analysis.

## 4. Conclusions and Future Trends

In this paper, we presented the sample treatment methods and analytical techniques used for the preconcentration and determination of PQ and DQ in biological samples. In the last 12 years, the sample treatment and determination methods for PQ and DQ progressed substantially. The mostly commonly used sample preparation methods include LLE, SPE, LPME, SPME, and MDSPE. As far as we are concerned, modified SPE has attracted more and more attention, especially MDSPE.

Different detection methods, including HPLC–DAD, ion chromatography–triple quadrupole mass spectrometry, MDSPE–HPLC, UPLC–HRMS, and other analytical methods, have been developed for the simultaneous detection of PQ and DQ in biological samples. Among those analytical methods, the most popular analytical methods are LC–MS. In particular, triple quadrupole MS has distinguished advantages, including accurate quantification, high sensitivity, and high selectivity, and it is widely used in the determination of PQ and DQ. HRMS detectors will perform more and more important roles for the detection of PQ and DQ in the future based on their high precision and sensitivity and their detection of unknown compounds. There is still a demand for enhanced simplicity, speed, selectivity, and sensitivity for PQ and DQ, and UPLC combined with HRMS detection is a promising analytical method. In addition, MALDI–FTICR–MS and SALDI–TOF–MS are becoming popular for their ultra-high resolution, high sensitivity and accuracy, low sample consumption, convenient preparation, speed, and ease of operation.

## Figures and Tables

**Table 1:** Comparison of reported pretreatment methods of PQ and DQ in biological samples.

Analytical Methods	Analytes	Matrix	Pretreatment Methods	Adsorbent/ Extractant	Recovery	LOD_S_	Ref.
UPLC–HRMS	PQ & DQ	Human urine	MSPE	Fe3O4@SiO2@poly(4-VB)	PQ: 86.7–104.3% DQ: 100.3–109.9%	PQ: 0.12 μg/L DQ: 0.14 μg/L	[33]
LC–MS/MS	PQ & DQ	Blood & urine	Protein precipitation	Acetonitrile	PQ: Blood: 87.32–94.96%/urine: 88.93–108.36% DQ: Blood: 80.28–91.54%/urine: 95.56–101.02%	PQ: 0.1 μg/mL DQ: 0.05 μg/mL	[14]
HPLC–HRMS	PQ & DQ	Blood	Protein precipitation	Acetonitrile: water = 3:1 (*v*:*v*)	PQ: 86–108% DQ: 88–96%	PQ: 5 ng/mL DQ: 10 ng/mL	[19]
HPLC–DAD	PQ & DQ	Human plasma	SPE	2 mL of ammonium formate, 2 mL of methanol, and 1 mL of a mixture solution of acetonitrile, ethyl acetate, and formic acid (4:4:2, *v*/*v*/*v*)	PQ: 95.38–103.97% DQ: 94.79–98.40%	0.01 μg/mL	[24]
HPLC	PQ & DQ	Plasma & urine	MDSPE	*CoFe_2_O_4_@SiO_2_ MNPs*	87.5–98.7%	PQ: 4.5 μg/L DQ: 4.3 μg/L	[35]
HPLC–UV	PQ	Biological samples	LLME	Mixture of triethylamine and water	90.0–92.3%	0.2 μg/L	[30]
HPLC–MS	PQ	Urine	DSPE	magnetic single-walled carbon nanotubes	92.89–108.9%	0.94 μg/L	[34]
GC–MS	PQ	Plasma & urine	HS-SPME	Polydimethylsiloxane fiber	Plasma: 94.00–99.85% Urine: 95.00–100.34%	0.01 μg/mL	[29]
UPLC–HRMS	PQ	Plasma & urine	Protein precipitation	Acetonitrile	Plasma: 98.54–100.90% Urine: 93.51–99.45%	Urine: 0.1 ng/Ml Plasma: 0.3 ng/mL	[13]
GC–MS	PQ & DQ	Serum & urine	Monolithic spin column extraction	0.2 mL of a mixture of chloroform and methanol (9:1 *v*/*v*) (MonoSpin^®^ C18 extraction column)	51.3–106.1%	PQ: 0.1 μg/mL DQ: 0.025 μg/mL	[26]
HPLC–MS	PQ & DQ	Human urine	SPE	1 mL of 5% methanol in deionized water (*v*/*v*) & 10% formic acid in acetonitrile (*v*/*v*) (Strata-X-CW 33 μm polymeric 3 mL weak cation cartridges)	PQ: 83.4–85.5% DQ: 77.7–94.2%	1 ng/mL	[27]
UV-Vis Spectrometry	PQ	Plasma & urine	MSPE	Fe_3_O_4_@SiO_2_ NPs	Plasma: 93.6–102.4% Urine: 92.9–103.5%	12.2 μg/L	[36]
UPLC–HRMS	PQ	human urine	MSPE	Amphiphilic carboxyl-functionalized magnetic polymer microspheres (Amphiphilic-MPs-COOH)	84.5–103%	0.1–1.6 μg/L	[32]
LC–MS	PQ	Tissue	Whirling agitated single-drop microextraction	1-dodecanol	>91.21%	4.81 ng/g	[31]
HPLC–UV	PQ	Human plasma	protein precipitation with organic solvent backwashing	Acetonitrile and methylene chloride	91.9%	0.01 μg/mL	[21]
HPLC–UV	PQ	The whole blood	Protein precipitation with hydrochloric acid	Acetonitrile	87.9–106.7%	0.026 μg/mL	[18]
HPLC–UV	PQ	Human plasma	Protein precipitation	Trichloroacetic acid-methanol (1:9)	n.d.	n.d.	[17]

n.d.: not found; LOD_S_: Limits of Detection.

**Table 2:** Examples of LC–MS and LC–MS/MS methods for the detection of PQ and DQ.

Matrix	Analytical Column	Mobile Phase	LODs	LOQs	Ref.
Blood/Urine	Agilent ZORBAX SB-Aq column (100 mm × 2.1 mm, 1.8 μm)	A: 15 mM HFBA B: acetonitrile Flow rate: 0.30 mL/min Gradient elution	PQ: 100 ng/mL DQ: 50 ng/mL	PQ: 200 ng/mL DQ: 100 ng/mL	[14]
Urine	Capcell Pak ST column (Shiseido Co., Ltd., Japan; 150 mm × 2.0 mm, 2.6 μm)	A: 0.4%TFA in water B: acetonitrile Flow rate: 0.30 mL/min Isocratic elution	PQ: 0.94 ng/mL	PQ: 2.82 ng/mL	[34]
Urine	SIELC Obelisc R columna (150 mm × 2.1 mm, 5 μm)	A: ammonium formate in water (Ph = 3.7) B: acetonitrile Flow rate: 0.5 mL/min Isocratic elution	PQ: 0.12 ng/mL DQ: 0.14 ng/mL	n.d.	[33]
DBS (Dry Blood Spot)	Hypersil GOLD C-18 column (100 mm × 2.1 mm, 1.9 μm)	A: 20 mM ammonium acetate with 0.1% formic acid B: acetonitrile Flow rate: 0.3 mL/min Isocratic elution	PQ: 0.5 ng/mL	PQ: 0.5 ng/mL	[43]
Plasma/Urine	ACQUITY UPLC bridged ethyl hybrid (BEH) HILIC column (100 mm × 2.1 mm, 1.7 μm)	A: 0.5% formic acid in 40 mM ammonium formate B: acetonitrile Flow rate: 0.3 mL/min Gradient elution	Plasma: 0.3 ng/mL Urine: 0.1 ng/mL	Plasma: 0.8 ng/mL Urine: 0.3 ng/mL	[13]
Plasma/Urine	Kinetex™ HILIC column (50 mm × 2.10 mm, 2.6 μm)	A: 250 mm ammonium formate containing 0.8% formic acid in water B: acetonitrile Flow rate: 0.3 mL/min Gradient elution	n.d.	PQ: 0.01 ng/mL	[20]
Plasma	Hypersil Gold C18 Column (250 mm × 4.6 mm, 5 μm)	A: acetonitrile B: 75 mmol/L sodium heptanesulfonate water solution (including 0.1 mol/L phosphoric acid) Flow rate: 1.0 mL/min Gradient elution	n.d.	PQ: 50 ng/mL	[23]
Plasma/Urine	Ion Pac CS18 (250 mm × 2.0 mm, 6.0 μm)	A: 30 mM MSA B: formic acid:acetonitrile (3:100, *v*:*v*) Flow rate: 0.3 mL/min Gradient elution	PQ: 1 ng/mL DQ: 0.5 ng/mL	PQ: 0.3 ng/mL DQ: 0.2 ng/mL	[22]
Urine	Atlantis^®^ HILIC Silica, (150 mm × 2.1 mm, 5 μm)	A: 250 mM ammonium formate in deionized water, pH 3.7 B: acetonitrile Flow rate: 0.4 mL/min Isocratic elution	PQ: 0.63 ng/mL DQ: 0.13 ng/mL	n.d.	[27]
Blood	Hypersil GOLD^TM^C18 (100 mm × 2.1 mm, 1.9 μm)	A: 0.1% formic acid B: methanol Flow rate: 0.3 mL/min Gradient elution	PQ: 5 ng/mL DQ: 10 μg/mL	PQ: 10 ng/mL DQ: 20 ng/mL	[19]
Brain Tissue	ZORBAX RX-C8 column (150 mm × 4.6 mm, 5 μm)	with a three-solvent system: 0.1% formic acid in water (A), 0.1% formic acid in methanol (B), 0.1% formic acid in acetonitrile (C) Flow rate: 0.3 mL/min Gradient elution	PQ: 0.1 ng/mL	PQ: 2 ng/mL	[31]

n.d.: not found.

**Table 3:** Comparison of different MS analyzers for PQ and DQ.

Single Q MS	QQQ MS	LIT–MS	TOF MS	Orbitrap MS	FTICR MS
Advantages:					
Relatively small size and low cost	Cascade function and strong qualitative ability	Multistage tandem MS	Faster scan speed than that of orbitrap	Wider dynamic range than TOF (4 order of magntude)	Capability of multi-level cascade
Robustness and ease of maintenance	Good quantitative ability	Higher sensitivity than that of traditional 3D IT	high mass upper limit (6000–10,000 u)	Fast positive–negative ionization switching	Very good qualitative ability
Commonly used, especially in GC–MS	Higher S/N than that of single Q MS	Durable and easy to miniaturize	Good resolution for high *m*/*z* ions and large molecules	Higher resolution than TOF MS	Highest resolution; best sensitivity compared with the other four MS analyzers
	Varied scanning modes (MRM, SRM, neutral loss, etc.)	Unknown components with a low content can be analyzed	High sensitivity	Stable mass axis (1 week) that is not affected by the environment	Capability of being combined with other ionization sources for the detection of different polarity compounds
Disadvantages:					
Low resolving power and disturbances from the isotope and the other *m*/*z* approximation ionic	Low resolution	Compared to QQQ, there is a lack of a characteristic group screening function	Mass axis needs to be calibrated frequently	Limited ion capacity	Bulk weight, high cost
Insufficient quantitative ability	High cost and the requirement of delicate maintenance compared single Q MS	Relatively low resolution	More expensive than Q MS	Incapability of the cascade alone	Limited data acquisition speed
Limited capability in terms of mass range (usually <4000 Da)	Lack of quantitative analysis ability for untargeted unknowns	Narrower range of quality analysis than that of TOF	Noisier TOF baselines than those of orbitrap due to spurious signals	Limited data acquisition speed in the high mass resolution setting	Maintenance is very expensive

**Table 4:** Examples of electrochemical sensors for the detection of PQ and DQ.

Matrix	Analytes	Electrode	Technique	Linear Range (M)	Detection Limit (M)	Ref.
Human urine, serum, natural samples	PQ	DNA-3D GNP/GE	DPV	7.0 × 10^−9^–1.5 × 10^−6^	2.0 × 10^−10^	[63]
Potato, lemon, orange, and natural water samples	PQ	CCPE	DPASV	5 × 10^−7^–240 × 10^−7^	1.63 × 10^−9^	[64]
Meconium	PQ	Ab/C60-FC-IL-GCE	CVs	3.89 × 10^−11^ to 4.0 × 10^−8^	9.0 × 10^−12^	[65]
Human urine and grain	DQ	CA DNA-GNP/GE	DPV	1.0 × 10^−9^ to 1.2 × 10^−6^	2.0 × 10^−10^	[77]
Apple and potato	PQ	FAP-CPE	SWV	5 × 10^−8^ to 7 × 10^−5^	3.5 × 10^−9^	[78]
Water and urine samples	DQ	Dq-PT-2-NPOE-Na-TPB/CPE	Potentiometric titration methods	3.8 × 10^−6^ to 1.0 × 10^−3^	9.0 × 10^−7^	[79]

DNA-3D GNP/GE: DNA-three-dimensional gold nanoparticles-modified gold electrode; CCPE: clay-modified carbon paste electrode; Ab/C60-FC-IL-GCE: electrochemical immunosensor (coated with Polyclonal antibody (Ab)) modified with a composite made from fullerene-C60 (C60), ferrocene (FC), and the ionic liquid; CA DNA-GNP/GE: unmodified DNA molecules with consecutive adenines (CA DNA) and gold nanoparticles (GNPs) were fabricated on gold electrodes; FAP-CPE: fluorapatite film carbon paste electrode; Dq-PT-2-NPOE-Na-TPB/CPE: diquat phosphotungstate dissolved in 2-nitrophenyloctyl ether as a pasting liquid with 1.0% Na-TPB as an additive; DPV: Differential pulse voltammetry; DPASV: differential pulse anodic stripping voltammetry; CVs: Cyclic voltammetries; SWV: Square wave voltammetry.

## Data Availability

The data presented in this study are available in the article.

## Abbreviation

APPI	atmospheric pressure photoionization
C4D	capacitively coupled contactless conductively detection
CE	electrophoresis
CE-DAD	capillary electrophoresis and diode-array detection
DBS	dried blood spot
DQ	diquat
ELISA	enzyme-linked immunosorbent assay
EPQ	ethyl paraquat
ESI	electrospray ionization
GC	gas chromatography
GC–MS	gas chromatography–mass spectrometry
HESI	heated electrospray ionization source
HFBA	heptafluorobutyric acid
HILIC	hydrophilic interaction liquid chromatography
HPLC	high-performance liquid chromatography
HPLC–UV	high-performance liquid chromatography–ultraviolet detection
HRMS	high-resolution mass spectrometer
HS-SPME	headspace solid-phase microextraction
ICA	immunochromatographic assay
IS–SERS	internal standard–SERS
LC/TOF–MS	liquid chromatography/time-of-flight mass spectrometry
LC–MS	liquid chromatography–mass spectrometry
LLE	liquid–liquid extraction
LOD	limits of detection
LOQ	limits of quantification
LPME	liquid-phase microextraction
MALDI–FTICR–MS	matrix-assisted laser desorption/ionization Fourier transform ion cyclotron resonance mass spectrometry
MCS	microcapillary sampling
MDSPE	magnetic dispersed solid-phase extraction
MODS	multi-organ dysfunction syndrome
MS	mass spectrometer
MSWCNTs	magnetic single-walled carbon nanotubes
NaBH4–NiCl2	sodium borohydride–nickel chloride
NP	nanoparticle
PAD	paper-based analytical devices
PFs	preconcentration factors
PQ	paraquat
PRM	parallel reaction monitoring
QqQ	triple quadrupole
SALDI-TOF MS	surface-assisted laser desorption/ionization time-of-flight mass spectrometry
SERS	surface-enhanced Raman spectroscopy
SHS	switchable–hydrophilicity solvent
SPE	solid-phase extraction
SPME	solid-phase microextraction
TEA	triethylamine
TRFICA	time-resolved fluorescence immunochromatographic assay
UA–SHS–HLLME	ultrasound-assisted switchable-hydrophilicity solvent-based homogeneous liquid–liquid microextraction
UPLC–MS	ultra-high-performance liquid chromatography–mass spectrometry
